# Values in evolutionary biology: a comparison between the contemporary debate on organic progress and Canguilhem’s biological philosophy

**DOI:** 10.1007/s40656-022-00493-z

**Published:** 2022-05-25

**Authors:** Silvia De Cesare

**Affiliations:** Department of Philosophy, Université de Genève, Rue De-Candolle 2, 1205 Genève, Switzerland

**Keywords:** Values, Philosophy of evolutionary biology, Georges Canguilhem, Organic progress, Normativity, Fitness

## Abstract

The aim of this paper is to make a comparison and build up a dialogue between two different philosophical approaches to values in evolutionary biology. First, I present the approach proposed by Alexander Rosenberg and Daniel McShea in their contribution to the contemporary debate on organic progress. i.e. the idea that there has been some kind of improvement concerning organisms over the history of life. Discussing organic progress raises the question of what “better” exactly means. This requires an explicit clarification on what legitimately means to speak about “good” in evolutionary biology, thus to speak about values. Second, I move on to present an approach to values that has been proposed by Georges Canguilhem in the context of a different philosophical tradition (i.e. the “continental” tradition). Canguilhem’s original theses are conceived in a Darwinian framework and clearly relate to the question of values in evolutionary biology. I shall then propose a comparison between these two heterogeneous perspectives on values by critically evaluating their common points and main differences. I will argue that both perspectives agree that the question of values in evolutionary biology takes on its full meaning with respect to the relationship between the organism and the environment. However, the framework for conceptualizing values in evolutionary biology provided by Rosenberg and McShea neglects a significant point highlighted by Canguilhem, i.e. the active role that the organism can play in evaluating the environment. In line with recent developments of biology (e.g. niche construction), this point can be easily integrated into Rosenberg and McShea’s framework. Finally, I will point out some main differences between the two perspectives relative to the specificity of Canguilhem’s biological philosophy.

## Introduction

The philosophical question concerning normativity is generally debated in the domains of moral, social and political philosophy, in aesthetics and in epistemology. In this paper, this question will be discussed with respect to the life sciences, and particularly evolutionary biology. The main question that I shall address concerns some statements in evolutionary biology where *values* clearly come into play. A broad definition of value could be: the value of something (X) is the worth that X has for someone (Y). This definition can be applied to statements where values are attributed to biological entities, referring in particular to “adaptive values” which make sense with respect to the theory of evolution by natural selection. For example, it can be affirmed that the coat (X) has a certain worth for a polar bear (Y), as well as the shell (X) has a certain worth for a clam (Y). In these examples the organic trait is “good” for the organism, but it may also be said that some feature has a negative value for a living being (for example, in a specific environment, a heavy shell could be a burden for the clam). The aim of this paper is to propose a philosophical understanding of this kind of axiological statements in evolutionary biology, by building up a dialogue between two different approaches about values.^1^

The first approach, presented in Sect. 2, is the one proposed by two authors contributing to the contemporary debate in philosophy of biology, Alexander Rosenberg and Daniel McShea. I will focus on their contribution to the debate on organic progress, i.e. the idea that there has been some kind of improvement concerning organisms over the history of life. Indeed, discussing organic progress presupposes specifying what “better” means, and this requires an explicit discourse about values.

In Sect. 3, I will discuss the approach of the French philosopher Georges Canguilhem (1904–1995), proposed in the context of the philosophical tradition generally labelled as “continental”. The question of values with respect to living beings is at the core of Canguilhem’s thought since the beginning of its philosophical reflection (Limoges, 2015). Although it would be hard to present an exhaustive account of this thought, some key points will be presented.

In Sect. 4, I shall propose a comparison between these two perspectives on values and critically evaluate their common points and main differences. In Sect. 4.1, I will argue that the main common point is the following: both perspectives agree that the question of values in evolutionary biology takes on its full meaning with respect to the relationship between the organism and the environment. However, the framework for conceptualizing values presented by Rosenberg and McShea (2008) neglects a point highlighted by Canguilhem, i.e. the active role that the organism can play in evaluating the environment. I shall note that Canguilhem’s perspective is in line with the work of other scholars that have emphasized the same point (Lewontin, 1983; Odling-Smee et al., 2003). I will claim that this point can be easily integrated into Rosenberg and McShea’s framework. Finally, in Sect. 4.2, I will highlight some main differences between the two perspectives, related to the specificity of Canguilhem’s biological philosophy.

## Understanding values in Rosenberg and Mcshea’s framework

Several authors in the analytical tradition have examined the question of normativity applied to living beings. For exemple, in metaethics, “natural goodness” is discussed in detail by Foot (Foot, 2010), even though earlier developments can be found in Thomson (1996) and Hursthouse (1999). However, I have chosen to focus on the contribution of Rosenberg and McShea (2008) because it deals specifically with evolutionary biology and raises the explicit question of what “good” can mean in this discipline. Their contribution is put forward in the context of the contemporary debate around the concept of organic progress. Thus, I will shortly introduce this debate before presenting and analysing their contribution.

### The question of organic progress

Progress is generally defined as a “change towards the better”, implying a descriptive and an axiological element (Goudge, 1961). The notion might be applied to human civilization, scientific theories, technical artefacts and living beings. Applied to living beings, this notion boils down to saying that there has been some kind of improvement in the history of life (or parts of the history of life). Several authors, particularly Ruse (1996), have shown that this idea is complexly interwoven with the history of evolutionary biology. As nicely expressed by the historian of science John C. Greene, “evolutionary biologists, it seems, can neither live with nor live without the idea of progress” (Greene, 1990, p. 55).

This ambiguous feeling of evolutionary biologists with respect to the idea of progress can be related to its axiological element: why would evolutionary change be “towards the better”? Starting from Darwin, this question has to be understood with respect to the mechanism of natural selection. Darwin’s reasoning runs as follows: “in one particular sense the more recent forms must, on my theory, be higher than the more ancient; for each new species is formed by having had some advantage in the struggle for life over other and preceding forms.” (Darwin, 1859, p. 337). Here, natural selection is considered to be the *cause* leading to somewhat “better” organisms. But the exact way in which we should understand this betterment puzzled Darwin himself, as well as a great number of twentieth century’s evolutionary biologists, namely the architects of the Modern Synthesis (Ruse, 1996). Still today, the question of the relationship between evolutionary theory and the idea of organic progress continues to raise debate in contemporary philosophy of biology (De Cesare, 2019; Nitecki, 1988; Rosenberg & McShea, 2008).

### What does “better” mean in biology?

Rosenberg and McShea (2008) have made a significant contribution to this debate by proposing a clarification of how the axiological element implied in the idea of progress should be understood. I shall here sum up their contribution in chapter 5 from their *Philosophy of biology. A contemporary introduction* (the chapter bears the title “Complexity, directionality and progress in evolution”).

In this chapter, Rosenberg and McShea clearly identify the main problem: if organic progress is a change towards the better, what does “better” mean in biology? Is it even a scientific concept? They start by identifying two main ways in which the evaluative component in the idea of progress could be understood:

Now there are two ways in which an increase in some feature of organisms might be valuable. The feature could be valuable to us, to human beings. *Or it could be valued by the evolutionary process, so to speak, valued in the sense of preserved or enhanced owing to its adaptive value, the contribution it makes to survival and reproduction.* The more progressive organism could be the one that is better at surviving and reproducing, the one that is more fit, so that progress would be an increase in whatever features of organisms underlie increased fitness, over the history of life. (Rosenberg & McShea, 2008, p. 128, italics added)

The first option—considering the feature to be valuable to us—is immediately identified as problematic from a scientific standpoint. This is because there is a general agreement on the fact that human values should not be relevant “to the findings of science or to the process of determining what is true” (Rosenberg & McShea, 2008, p. 129). This is the idea of value neutrality of science.

Rosenberg and McShea propose to focus on the second option, “valued in the sense of preserved or enhanced owing to its adaptive value”. Here they introduce an important distinction between “intrinsic” and “instrumental” values. The difference is the following: if we say that X is better than Y in order to achieve the purpose Z, the word “better” is being used instrumentally. The technical example they present concerns microscopes: “an electron microscope is better than a light microscope for the purpose of seeing the very small details of certain objects”. (Rosenberg & McShea, 2008, p. 129). Here “better” refers to the specific purpose of seeing very small details; the question is not that an electron microscope is intrinsically valuable on its own. Rosenberg and McShea define the judgments of intrinsic values as “normative”, and they consider them to be a matter of moral or ethical value. They argue that, in virtue of value neutrality, science should not assert or deny the existence of intrinsic values. By contrast, science is not precluded from identifying the instrumental values of things, indeed instrumental values “are essential” (Rosenberg & McShea, 2008, p. 129).

Rosenberg and McShea apply this idea to a biological case:

Switching to a biological example, the theory of natural selection identifies the thick coat of the polar bear as instrumentally valuable for insulation, and insulation as instrumentally good for survival and reproduction. But science stops at this point. It does not identify some further good for which survival and reproduction are instrumentally valuable. Nor does it suggest that survival and reproduction are intrinsically good in themselves. It is silent on this matter owing to its value neutrality. (Rosenberg & McShea, 2008, p. 129)

As in the case of microscopes, we are referring to instrumental values when we say that the coat of the polar bear is good for insulation, and that insulation is good for survival and reproduction. Rosenberg and McShea clarify this point: statements of instrumental values are subject to empirical test, once we stipulate the criteria of goodness we have in mind – for example efficiency, speed or cost. By contrast, no test or experiment would settle disputes about statements of intrinsic values (Rosenberg & McShea, 2008, p. 130).

### Analysis of Rosenberg and McShea’s framework

Now that the framework has been presented, I shall move to analyse and make explicit some of its elements. To this end, three remarks may be useful.

As a first remark, I shall point out that the causal process responsible for preserving “valuable” organic entities is the mechanism of natural selection. Rosenberg and McShea affirm that organic entities are “valued by evolutionary process, so to speak”. To clarify what this statement means it shall be noted that, in the standard Darwinian framework, the selection is done by the environment. The environment is considered in its biotic components (predators, competitors, etc.) and abiotic components (temperature, oxygen, etc.). The metaphorical expression affirming that the environment “evaluates” thus means that the environment is a causal factor in preserving or enhancing some organic entities.^2^ This preservation or enhancement takes place *in virtue of* the adaptive values of organic entities. In the standard Darwinian framework, the basis is organic variation: some organic entities happen to be “good” with respect to a given environment, thus they are preserved.

As a second remark, I shall note how this framework answers the question: *what has an (adaptive) value?* This question refers to what is the ontology of interest, i.e. the organic entities considered to have a value. From the examples given by the authors, one can note that “having a value” can be predicated of different ontological levels.

- It can be predicated of *parts* below the level of the organisms, as in the case of the morphological organic trait “thick coat” (or it could be the wing of a bird, or the shell of a clam).
- It can be predicated of *whole organisms*: the polar bear (or the bird, or the clam) can be considered *fit* in a given environment.

In case (a), the underlying idea is that the organic trait has a value for a function, e.g. the function of insulation. The trait is considered as an *adaptation* for a *function*. In case (b), the reference is to the concept of *fitness*, generally intended as survival and reproduction (I shall note below the problems raised by this notion). Thus, we may say that some organic entities (parts or whole organisms) happen to be “good” *relative to* a given environment. In virtue of this “goodness”, they are preserved in the evolutionary process. I shall note that, in the biological discourse, evolutionary biologist might also consider other ontological levels as having an adaptive value, for example genes. One may also ask whether having a value can be predicated about levels above the organisms, e.g. populations or species. Finally, the question “*what has an (adaptive) value?”* refers to the complex problem in the philosophy of biology that is known as the question of units or levels of selection.^3^

My third remark concerns a point that clearly appears in Rosenberg and McShea’s framework: the question of values in evolutionary biology has at its heart the problematic notion of *fitness*. The authors affirm that “progress would be an increase in whatever features of organisms underlie increased fitness, over the history of life” (Rosenberg & McShea, 2008, p. 128). Saying that an organic entity is *fit* seems to refer to the “goodness” of the relationship between the organic entity and the environment. But what does “good” mean here and how to characterize it? In other words, what is *fitness*?

This term has a weighty semantic charge^4^ and philosophers of biology have pursued in vain an uncontroversial definition. As Rosenberg and Bouchard (2021) put it, understanding the meaning of the expression “survival of the fittest” is still of philosophical and biological urgency today. A complication is that we are here confronted with two interwoven problems: *defining* fitness and *measuring* fitness. As Rosenberg and McShea point out, these two aspects must be conceptually separated. They suggest a useful analogy to illustrate this idea: seconds and years measure time, but they do not define it (Rosenberg & McShea, 2008, p. 54). Ideally, we would like to provide a definition of “fitness” that easily lends itself to measurement, thus making quantitative explanation and prediction possible. (Rosenberg & McShea, 2008, p. 55). While it is here impossible to fully tackle this complex issue, it may be useful to give a sketch of the debate by distinguishing two possible concepts of fitness: ecological fitness and evolutionary (or Darwinian) fitness.

“*Ecological fitness*”—A first concept, that may be called “ecological fitness” (Rosenberg & Bouchard, 2021) refers to the “correspondence” between organismal traits and various aspects of the environment in which the organism is living. Here the question concerns the idea of appropriate organism design, e.g. if we ask what makes that, in a species of bird, the wing X is *fit* for the kind of flight Y (Gayon & Petit, 2018, p. 293). Note that this concept does not need to invoke evolution, as the question rather concerns functional morphology (Gayon & Petit, 2018, p. 293). Rosenberg and McShea seem to refer to a similar concept when discussing the possibility of defining fitness by invoking the notion of solving environmental design problems (Rosenberg & McShea, 2008, p. 55). However, they also acknowledge that this definition is difficult to convert into measurements.

“*Evolutionary (or Darwinian) fitness*”—The second concept of fitness, that may be labelled as “evolutionary” or “Darwinian”, is rather referring to the reproduction of the organism, and not only to its survival in the environment. The difference with ecological fitness is that we have here an evolutionary concept, where the focus is rather on the level of populations. This definition seems to easily lend itself to measurement via the counting of the offspring. This evolutionary or Darwinian concept is the one that has been central in population genetics starting from Ronald Fisher, who transformed the qualitative concept of fitness in a measure including mortality and fecundity rates (Gayon & Petit, 2018, p. 248). The focus here shifts from the organism to the contribution that the organism gives to form future generations. However, the fact that measures of Darwinian fitness are widely used does not mean that they are not problematic. How to “count offspring” might, indeed, not be straightforward in every possible environment. As Rosenberg and McShea point out:

“Sometimes a better measure of fitness is given by counting only male offspring or female offspring or grand-offspring, or granddaughters or grandsons. Sometimes the fittest will be those who have fewer offspring in a harsh season and more offspring in a lush one. Which will be the “right” measure depends on the environment in which organisms find themselves.” (Rosenberg & McShea, 2008, p. 55).

A possibility to modify this evolutionary concept of fitness—notably proposed by Brandon (1990)—is to adopt a “probabilistic propensity” definition of fitness. A propensity is something that an object has but manifests only in some circumstances (such as being magnetic) (Rosenberg & McShea, 2008, p. 56). For fitness, this would mean: X is fitter than Y in environment E if X has the probabilistic propensity to leave more offspring than Y in E. The idea behind this definition is that the most probable outcome does not necessarily correspond to the actual outcome: the fit organism might “unluckily” not reproduce (Rosenberg & Bouchard, 2021). But Rosenberg and McShea point out that this definition is also problematic: how to measure the probabilistic propensity of a species to leave descendants in a way which is independent of the actual number of descendants it leaves? (Rosenberg & McShea, 2008, p. 57). This brief sketch of the debate over fitness hints at the complexity of these problems, which do not seem to have an uncontroversial solution at present.

To conclude this analysis, I shall say that my remarks concerning Rosenberg and McShea’s framework lead to two conclusions. First, the question of values in evolutionary biology takes on its full meaning relative to the relationship between organic entities and their environment. Second, the question of values refers to some huge open problems in the philosophy of evolutionary biology, namely the debate about levels of selection (remark 2) and fitness (remark 3). Even if the move of considering only instrumental values (putting aside intrinsic values) seems to simplify the issue, these questions remain open.^5^ I shall now try to show that these same questions can be found in Canguilhem’s biological philosophy, albeit in another form and different terms.

## Canguilhem’s biological philosophy

In this section, I will present another perspective on values in the life sciences, the one proposed by Georges Canguilhem. His work presents three aspects that are difficult to separate: medical philosophy, history of biology and philosophy of science. Jean Gayon observes that if the first two aspects are relatively well known, “in terms of the philosophy of science, and especially the philosophy of life sciences, until recently Canguilhem was almost completely unknown among American scholars” (Gayon, 1998, p. 305). Indeed, Canguilhem can hardly be considered as participating in the debate in the domain of the “philosophy of biology”, referring to the Anglo-Saxon tradition starting from the late 1960s; he rather used the expressions “biological philosophy” or “philosophy of life sciences” to characterize his own work (Gayon, 1998). However, several scholars have recently highlighted the fecundity of Canguilhem’s philosophical thought. The book *Vital Norms: Canguilhem’s The Normal and the Pathological in the Twentieth Century* (Méthot and Sholl 2020) is particularly interesting with this respect. The collection of contributions proposes fresh perspectives to read Canguilhem’s work by exploring its historical, philosophical, and social dimensions.^6^

Although it will not be possible to give an exhaustive presentation of Canguilhem’s rich contribution to philosophy of life science, I shall here present some key points concerning the centrality of the question of values in his thought.

### Main thesis of the *Essay on some problems concerning the normal and the pathological* (1943)

Very early in his work, the question of values and norms appears to be central in Canguilhem’s philosophical thought (Limoges, 2015, p. 34). In his book *Essay on some problems concerning the normal and the pathological* (1943), Canguilhem questions the categories of “normal” and “pathological” in the philosophy of medicine, claiming that they cannot be considered as descriptive but rather as axiological. Canguilhem shares this thesis with the German neurologist and psychiatrist Kurt Goldstein (1878–1965): the concepts of normality and illness require the concept of “individual being”.^7^ In his book from 1934, Goldstein claims that health, illness and recovery must be understood with reference to the notion of a personal individual norm (Goldstein, 2000).

A clinical argument (Gayon, 1998) is advocated by Canguilhem to support the same thesis as Goldstein in the field of philosophy of medicine: illness cannot be considered as a deviation from the statistical norm, but as the emergence of a new individual norm. The point is that, in this new norm related to the condition of illness, the individual is much more fragile with respect to its environment: the capacity to tolerate “the inconstancies of the environment” are reduced (Canguilhem, 1991, p. 197).^8^ The individual in a pathological state is able to live in a specific environment, but this environment is narrowed if compared to the healthy individual. In fact, the healthy individual is “more than normal”, it is “normative”. The concept of “normativity”, at the core of Canguilhem’s biological philosophy, does not only apply to human beings, but to living beings in general.^9^ In the *Essay*, normativity is referred to as an expression of the “dynamic polarity of life” (Canguilhem, 1991, p. 127), the main idea being that the living is never indifferent to its life conditions:

“Life is far removed from such an indifference to the conditions which are made for it; life is polarity. The simplest biological nutritive system of assimilation and excretion expresses a polarity. When the wastes of digestion are no longer excreted by the organism and congest or poison the internal environment, this is all indeed according to law (physical, chemical, etc.) but none of this follows the norm, which is the activity of the organism itself. This is the simple fact that we want to point out when we speak of biological normativity.” (Canguilhem, 1991, p. 127–128).

Thus, biological normativity can be understood as the ability of the organism to produce or establish a way of living in a given environment (Sholl, 2020). The normative organism has a flexible relationship to the environment and it is capable of tolerating the potential “inconstancies of the environment” (Canguilhem, 1991, p. 197). In contrast, the pathological organism expresses another kind of norms of life: “these norms are inferior to specific earlier norms in terms of stability, fecundity, variability of life” (Canguilhem, 1991, p. 197).^10^

### The role of evolution and natural selection in Canguilhem’s reasoning

As pointed out by Jeler (2014) and Méthot (2020) it is striking that, to discuss the categories of normal and pathological, Canguilhem always refers to the question of the evolution of species and the concept of natural selection. What is the link between the thesis that the pathological cannot be defined as a deviation from statistical norm and evolutionary issues? This challenging question has been tackled by Jean Gayon (Gayon, 1998) and more recently by Pierre-Olivier Méthot (Méthot 2020).^11^

The main point that interests Canguilhem is that, in the Darwinian theory, organic variability is at the very basis of the evolutionary process. In a population, between all possible variations, some organisms may present mutations that are deviations from the statistical norm. Already in the forties, when writing the *Essay*, Canguilhem was already interested in the experimental work on natural selection by the French biologist Georges Teissier. Canguilhem finds in Teissier’s work the empirical confirmation of the fact that statistical anomalies (deviations from statistical norms) should not be regarded as pathological from the outset. In some contexts, where the mutation happens to be viable, statistical anomalies could be at the origin of a new species. This can be easily understood from the *Drosophila* experiences performed by Georges Teissier and Philippe l’Héritier. The two biologists have observed that certain mutations, which can seem disadvantageous in a species’ environment, can become advantageous if the conditions vary. In a sheltered and closed environment, *Drosophila* with vestigial wings are wiped out by *Drosophila* with “normal” and functional wings. But Teissier and L’Héritier created experimental conditions in an open and windy environment, in which *Drosophila* with vestigial wings “do not fly, feed constantly, and in three generations we see sixty percent vestigial *Drosophila* in a mixed population” (Canguilhem, 1991, p. 142). Thus, *Drosophila* with vestigial wings become “normal” in the new environment. The notions of normality and abnormality are meaningful only with respect to the relationship between the organism and the environment. Twenty years after the *Essay*, in the “New Reflections on the normal and the pathological”, Canguilhem would be still reasoning on these issues and defining the norm as “the kind of deviation that natural selection maintains”.^12^

### Organic variability and the question of values

How does this reasoning on evolution and natural selection relate to the question of values? This question is clearly answered, I think, in the text from 1951 “The normal and the pathological”, published in *The Knowledge of Life* (Canguilhem, 2008). As Gayon (1988) remarks, the key point is the inseparability between the notion of *organic variability* and the problem of biological value.

“Individual singularity can be interpreted either as a failure or as an attempt, as a fault or as an adventure… as attempts or adventures, living forms are considered not beings referable to a real, pre-established type but organizations whose validity (that is, value) must be referred to the eventual success of their life.” (Canguilhem, 2008, p. 125).

Here Canguilhem claims that the “value” of an organic form must be referred to the eventual success of their life. He also remarks, a few lines later, that there is an identity between “value” and “health” (in Latin, *valere* means “to be well”). However, the success of an organic form cannot be anticipated a priori and it is always temporary and threatened:

“There is no in itself a priori difference between a successful form and a failed form [forme manquée]. Properly speaking, there are no failed forms. Nothing can be lacking [manque] to a living being once we accept that there are a thousand and one different ways of living. Just as in war and politics there is no definitive victory, but only a relative and precarious superiority or equilibrium, so in the order of life there are no successes that radically devalorize other attempts and make them appear failed. All successes are threatened, since individuals and even species die. Successes are delayed failures; failures are aborted successes. What decides the value of a form is what becomes of it.” (Canguilhem, 2008, p. 126).

If the value of an organic form is its temporary “success”, what is the connection between values and natural selection? Selection is the sorting or screening done by the environment:

“…We must note that different genotypes—the lineages of a given species—present different “values” in relation to ambient circumstances. Selection, that is, screening by the milieu, is sometimes conservative in stable circumstances and sometimes innovative in critical circumstances. At certain times, "the riskiest attempts are possible and licit."” (Canguilhem, 2008, p. 127).

The relationship between the concept of natural selection and question of values is still discussed in a text published in 1977 (“The question of normality in the history of biological thought”), where Canguilhem analyses the work of Darwin. Canguilhem claims that, even if the mechanism of natural selection is non-teleological, this does not mean that Darwin has eliminated from the idea of life every reference to a “comparison of values” (Canguilhem, 2009, p.166, my translation). The sorting by natural selection gives, as a result, the adaptation of organic beings. If Darwin has detached the concept of “adaptation” from that of a preordained purpose, he has conceived adaptation with respect to “normality”.^13^ The result of a non-teleological mechanism are beings whose life is “a difference of value with respect to death”.^14^ The normality of a living being is “that quality of its relation to the environment that enables it to generate descendants exhibiting a range of variations and standing in a new relation to their respective environments, and so on.” (Canguilhem, 1988, p. 137).^15^

### The active role of the organism with respect to the environment

In Sect. 3.3, I have discussed organic variability meaning with this term “inter-individual” variability. In that sense, natural selection can be seen as sorting out individuals that have inherited valuable variations^16^: this would suggest that the environment *determines* the organisms that will be viable. But an additional feature, central to Canguilhem’s reasoning, introduces more complexity to this picture: the organism cannot be considered as being merely passive with respect to the environment. This is clear for Canguilhem already in the *Essay*, where he states in the conclusion:

“Types and functions can be qualified as normal with reference to the dynamic polarity of life. If biological norms exist it is because *life, as not only subject to the environment but also as an institution of its own environment, thereby posits values not only in the environment but also in the organism itself*. This is what we call biological normativity.” (Canguilhem, 1991, p. 227, italics added).

This is developed in 1946–47 with Canguilhem’s idea of the organism as “center of reference” of values, which appears clearly in “The living and its milieu”. An organism, whether we are talking about a man, a plant or an amoeba, is never indifferent to its life conditions, it appreciates what is good or bad for itself (e.g. some physical condition, some food resource).

“Biology must first hold the living to be a significative being, and it must treat individuality not as an object but as an attribute within the order of values. To live is to radiate; it is to organize the milieu from and around a center of reference, which cannot itself be referred to without losing its original meaning”^17^(Canguilhem, 2008, p. 113–114).

An excerpt from “The living and its milieu” is particularly helpful to clarify this question of values relating to both organisms and the environment. While discussing the differences between the notion of environment in Lamarck and Darwin, Canguilhem writes:

“In any case, for Darwin, to live is to submit an individual difference to the judgment of the ensemble of living beings. This judgment has only two possible outcomes: either death or becoming oneself part of the jury for a while. So long as one lives, one is always judge and judged.” (Canguilhem, 2008, p. 105).

It is worthy to conceptually distinguish this double aspect of the organism being both “judge” and “judged”. For Canguilhem, so long as an organism live, it is *judged* (by the environment) but at the same time it *judges* by reacting actively to it. *The organism judges* because it is never indifferent to its environment and it assesses (although unconsciously, except in the case of man) what is positive or negative for its life. There is a “debate” between the organism and the environment, “to which the living brings its own proper norms of appreciating situations, both dominating the milieu and accommodating itself to it.” (Canguilhem, 2008, p. 113). This debate takes the form of a strong opposition for the pathological individual, whereas for the healthy individual it seems to be somehow a “soft” opposition.^18^ So long as the organism succeeds in coping with the environment (whether in a healthy or pathological situation, the point being to stay alive), it is also to some degree “member of the jury”. Indeed, the organism will be part, for a while, of the organic component of the environment (for example, we can imagine the case of an organism X acting as a competitor for an organism Y). At the same time, *the organism is “judged”.* The norms of the organisms are “sorted” by the “environment”: natural selection is understood both with its organic and inorganic components. In the experiments of Teissier and L’Héritier, *Drosophila* are “judged” by abiotic conditions (windy or sheltered). However, the “judgement” could also come from other organisms being members of the jury (competitors, predators, etc.) Although it is useful to distinguish them conceptually, these two aspects of “judge” and “judged” seem to be conceived by Canguilhem as two sides of the same coin. Ultimately, a physiologically well-adapted organism that can cope with its environment will be “positively sorted” in the evolutionary process.^19^

A last point is worthwhile to mention with respect to the idea of the organism not being passive with respect to the environment. As Sholl (2020) has pointed out, Canguilhem not only considers the organism as being “constructive” with respect to the environment, but also as presenting a certain “plasticity”. The idea is that the problem of biological variability has to be understood not only with respect to inter-individual variations, but also with respect to intra-organismic variability (see Sholl, 2020). Variability within the individual may be expressed through developmental or phenotypic plasticity or, in some organisms, by varying their behaviours. For Canguilhem variability is always “good”, because it is the only thing that guarantees the “promptness” of the living in reacting to the possible changes of the environment. In the *New reflections on the normal and the pathological*, Canguilhem also notes that this is also true for variability considered at a more general scale. In fact, if we consider the whole of living beings “in the continuity of life”, we see that there is a “*variation of modes of life* for the occupation of all vacant places” (Canguilhem, 1991, p. 263). Thus, variability can be regarded as “good” at least at three different levels: at the level of the organism (developmental or phenotypic plasticity, behaviour), at the level of the lineage (inter-individual variability) and at the level of “modes of life” (variability of ecological strategies).

## Comparison and critical perspective

The two perspectives presented here on the question of values with respect to the life sciences are clearly heterogeneous in their philosophical approach. However, they can be meaningfully be compared because the same problems lie behind the different styles and terminology. In Sect. 4.1, I shall first try to show the main point shared by the two perspectives and claim that Canguilhem’s argument on the active role of the organism can be considered as a complement of the framework of Rosenberg and McShea. Finally, in Sect. 4.2, I will highlight some differences between the two perspectives relative to the specificities of Canguilhem’s biological philosophy.

### “Good” makes sense with respect to the organism/environment relationship

The main point that is common to the two perspectives is that the question of values in evolutionary biology takes on its full meaning with respect to the relationship between organic forms and their environment. In both cases, this relationship is conceived in a Darwinian framework: this means that the mechanism of natural selection plays a central role in the evolutionary process. In other words, the environment (abiotic and biotic) plays a causal role in preserving and enhancing organic forms happening to be “good” or “fit”. It is possible to look at this process in a “deterministic” way, with the environment having a causal role and the organism passively going through the sorting. However, the fact that the organism has an active role in “carving out” the environment undermines this interpretation. Thus, instead of “happening to be good” relative to the environment, the organism has some room for manoeuvre for “becoming good” by modifying itself and/or its own environment. This point, central for Canguilhem, is not discussed in the chapter of Rosenberg and McShea.

It shall be noted that this emphasis on the active role of the organism, put forward by Canguilhem in his writings from the 1940’s onward, is in line with the subsequent developments of evolutionary biology.^20^ With this respect, a central paper is the one by Richard Lewontin in 1983: “The organism as the subject and object of evolution”. The author stresses the fact that the living being is not “acted upon” by an autonomous environment, “on the contrary, the environment and the organism actively codetermine each other” (Lewontin, 1983, p. 89).

“Organisms are the objects of the force of natural selection. This force sorts out the form that is the best solution to the problem posed by the external world. The word “adaptation” reflects this point of view, implying that the organism is molded and shaped to fit into a pre-existent niche, given by the autonomous forces of the environment, just as a key is cut and filed to fit into a lock.” (Lewontin, 1983, p. 98).

Lewontin points out the difficulties related to this view, both conceptual and factual. The fact that the organism constructs its environment needs to be incorporated by evolutionary theory. “The organism influences its own evolution, by being both the object of natural selection and the creator of the conditions of that selection” (Lewontin, 1983, p. 106).

As Sholl (2020) remarks, Lewontin’s arguments are increasingly used to explain some “neglected” aspects of evolutionary theory, particularly in the literature concerning “niche construction” (Laland et al., 2007; Odling-Smee et al., 2003). Sholl (2020) also points out the fact that Canguilhem’s ideas on normativity can be fruitfully compared with the research on developmental plasticity (see also West-Eberhard, 2005).^21^

Finally, the analysis of values put forward by Rosenberg and McShea can be easily integrated with this idea of the active role of the organism with respect to the environment.^22^ The key point to be acknowledged is that not only the environment has a causal role in preserving “good” (“fit”) organism, but also that the organism appreciates what is “good” for its life and might modify itself and/or the environment to favour it.

### Some differences related to the specificities of Canguilhem’s biological philosophy

Two main differences can be tentatively identified between the perspectives on values considered here. The first concerns the possibility of a quantitative approach to “goodness”, the second refers to the understanding of the concept of value relative to the dichotomy intrinsic/instrumental.

Let’s start from the first point. In the debate in the philosophy of biology, the “good” organism/environment relationship refers to the concept of “fitness”; in Canguilhem’s biological philosophy, this same relationship refers to the concept of “normativity”. Are these two concepts somehow overlapping? In both cases, the two aspects of survival and reproduction seems to be present. The notion of “ecological fitness” seems to be more related to the survival of the organism, whereas the notion of “evolutionary fitness” also includes reproduction (I have discussed these two notions in Sect. 2.3). On Canguilhem’s side, when speaking of a healthy organism as a normative one, the emphasis seems rather to be on the survival of the organism and its “flexible” relation to the environment. However, in several occasions, Canguilhem notes that the notion of normativity includes fertility: “for us a living species is viable only to the extent that it shows itself to be fecund, that is, productive of novelties, however imperceptible these may be at first sight.” (Canguilhem, 2008, p. 125). Fecundity is a way of producing variability, and variability is always “good”, because it is the only thing that guarantees the “promptness” of the living in reacting to the possible changes of the environment.^23^

Thus, survival and reproduction seem to be present both in the concept of evolutionary fitness and in Canguilhem’s concept of normativity. However, we may ask whether Canguilhem would have agreed on the fact that the “goodness” of the organism/environment relationship can be quantified. On their side, Rosenberg and McShea acknowledge the problems related to definition and measure of fitness, but seem to consider that a good definition of fitness should lend itself easily to measurement (so that it is worth trying to quantify the “goodness” of the relationship).^24^ My feeling is that Canguilhem would have been probably sceptical towards the attempts to objectivize and measure the “goodness” of a relation that is qualitative and dynamic. At least this is the conclusion of the *Essay* with respect to the categories of normal and pathological: “the concept of norm is an original concept which, in physiology more than elsewhere, cannot be reduced to an objective concept determinable by scientific methods.” (Canguilhem, 1991, p. 228). Ultimately, it is the individual patient who can tell if he/she considers a biological phenomenon as pathological or not: there seems to be no way to objectivize this qualitative feeling.

Finally, let us consider the second difference between the two perspectives on values, which concerns the stance that Canguilhem would have taken with respect to the distinction between instrumental and intrinsic values, as considered in Rosenberg and McShea’s chapter. The point is quite subtle, because probably Canguilhem would not have adhered to this dichotomic distinction.

Even though the exact significance of “intrinsic” is uneasy to grasp, it is generally meant that something having an intrinsic value has a value “in itself”, in virtue of its own properties and in a non-relational way (Moore, 1922). Values of this kind are seemingly not what Canguilhem refers to when he speaks of vital values. Indeed, he does not claim that organisms have an intrinsic value that would be given by the simple fact of being alive. Canguilhem stresses the difference between “existence” and “value”: “existence does not have value, it is not value”.^25^ In characterizing the opposition between the two terms, Canguilhem refers to the notion of “need”, which is tightly related to values. The main idea, developed in some unpublished texts from 1943, is that “need creates value”^26^: the organism “feels” something in its environment as having a negative value. Canguilhem takes the example of a plant that is exposed to heat. The mere fact of desiccation can be conceived on the plan of existence, but for the plant it is felt (“éprouvé”) as a need to conserve water. This feeling thus prompts the organism to react through physiological regulation (Limoges, 2015, p. 36). Canguilhem insists on the idea that a “need” is impossible to understand from an objective standpoint.^27^ The fact that the organism is feeling a need is an expression of the polarity of the living: “values are at the level of the polarity: they are the polarity itself”.^28^ Thus, the organism does not have any intrinsic value but it is somehow “source” of values, in the sense that it is producing or creating them. This creation of values takes place in the *relation* between the organism and the environment, relation in which the organism plays an active role.

The dichotomy presented by Rosenberg and McShea (2008) is between intrinsic and instrumental values.^29^ If the notion of intrinsic values does not seem to be relevant in Canguilhem’s perspective on vital values, this does not mean that he only admits instrumental values. He explicitly refers to this question while discussing specifically human values. In fact, in this paper I have focused on what Canguilhem calls “vital values” common to all living organisms, whether they are plants, amoebas, hedgehogs or human beings. For the human species, however, other values are important besides vital values: technical values, critical values (scientific, aesthetic) and philosophical values (moral, metaphysical).^30^ In a text where Canguilhem discusses the value of philosophy, he makes two interesting remarks on instrumental values, i.e. values to be understood exclusively in terms of “utility”. A first remark is that “it is not exact to say that every value is a value of utility, because utility strictly speaking is only the value of a means”.^31^ A second remark is that it is common that human beings are first interested by the means, without seeing that means only exist with respect to ends.^32^ These two remarks suggest that Canguilhem admits “non-instrumental” or “final” values as far as specifically human values are concerned.

Do these two remarks also apply to vital values, i.e. the values at stake in evolutionary biology? My interpretation is that Canguilhem would admit non-instrumental values also in that case, insofar as the organism (debating with its environment) might be seen as an endpoint of value attribution. In order to clarify this interpretation, let us consider the following claim: “biocarbonates composing the mollusc’s shell are instrumentally good”. They are good because they contribute to the strength of the shell, which is instrumentally good for defence. The defence function is itself instrumentally good for the mollusc to deal with its environment. However, there seems to be here an endpoint in the possibility of attributing instrumental values. This endpoint is the organism debating with the environment, which might be conceived as the “general function” of every living being (Canguilhem, 2008, p. 9). Thus, I suggest that we should interpret Canguilhem as acknowledging the existence of non-instrumental vital values, although these should not be understood as “intrinsic”.

## Conclusion

In this contribution, I tried to build up a dialogue between two different philosophical approaches to values with respect to evolutionary biology, namely the contribution of Rosenberg and McShea in the debate on organic progress and Canguilhem’s biological philosophy. Despite their divergences in terminology and philosophical style, these perspectives are led by common questions on values in life sciences. This is mainly due to the fact that they both understand values within the framework of a Darwinian view of life, where the living is considered in its dynamic relationship with the environment. “Fitness” and “normativity” are the two concepts that refer to the “goodness” of the relationship between the living and the environment. In both perspectives, natural selection (the causal action of the environment) is considered as a mechanism responsible to the preservation and enhancement of “good” livings relative to a given environment. I pointed out that Canguilhem made an important claim: the causal relation is not unidirectional (the environment determines the organism), but rather there are complex feedbacks between the two relata. In this co-construction, the organism is also active in “becoming good”. I argued that Canguilhem was on the right track and that the relationship between the organism and the environment should be regarded as bidirectional. Indeed, this is in line with recent developments of evolutionary biology. In their contribution to the debate on organic progress, Rosenberg and McShea neglects to discuss the active role played by the organism. However, I argued that this significant point can be easily integrated in their framework.

The fruitfulness of the comparison also lies in the challenging questions it has brought to the fore. How do we exactly define the “goodness” of a relationship between a living being and the environment? If a definition applicable to all living beings is possible, would this allow to quantify this qualitative relationship? Is there a privileged organic level (organism? lineage?) relative to which this “goodness” should be understood? While these questions remain open at present, I hope that this analysis has contributed to identify them and show their centrality for a philosophical understanding of values in evolutionary biology.

## Footnotes

1 The exact relationship between the terms “normativity”, “values” and “norms” is not easy to disentangle. The term “normativity” is generally used in the literature to encompass both “values” and “norms”, often considering these two terms as synonyms (Ogien and Tappolet 2009). In the ethical domain, Von Wright (1963) has proposed a distinction between value-concepts—the most important being “good” and “bad”—and normative concepts like those related to expressions such as “ought”. I have here chosen to speak mainly about “values”, because my main interest is to understand in which sense it is legitimate to speak of “good” in evolutionary biology. However, I acknowledge that the question of values and the question of norms are deeply interwoven and the discussion of Canguilhem’s work (Sect. 3) will be also referring to “biological norms*”.*

2 If we consider the definition of value given in the introduction – the value of something (X) is the worth that X has for someone (Y) – we see why in that case the expression “the environment evaluates organisms” is only metaphorical. The environment is not “someone” (even though it might include some organisms in its biotic components), so it is not possible to literally speaking of values with its respect.

3 See for example Sober (2006), Hull and Ruse (2007) and Lloyd (2020). For the distinction about value attributions to parts and whole organisms, see also De Cesare (2019).

4 See Gayon and Petit (2018), p. 291–292.

5 As far as I know, there have not been specific answers to the treatment of values proposed by Rosenberg and McShea (2008). Although not explicitly framed in terms of analysing values, the debate on organic progress is still open in philosophy of biology (see for example Rosslenbroich 2014).

6 For other recent contributions on Canguilhem, the reader may refer to the dossier “Canguilhem et la biologie” published in *Revue d’Histoire des Sciences* (see Loison 2018) and to the works of Elden (2019) and Talcott (2019).

7 It is generally admitted that Goldstein’s work had a huge influence on Canguilhem’s thought. Nevertheless, as noted by Limoges (2015, p. 37), Canguilhem has read Goldstein’s work quite late, when his main thesis about normativity was already formulated. About Canguilhem and Goldstein, see also Wolfe (2015) and Moya Diez (2018).

8 Here “environment” translates the term “milieu”. About the terms “enviroment” and “milieu”, see the essays “Aspects of vitalism” and “The living and its milieu” in Canguilhem (2008).

9 For detailed analyses of the complex concept of “normativity”, the reader may refer to Giroux (2010), Debru (2015), Moya Diez (2019), Méthot (2020) and Sholl (2020).

10 “The pathological is not the absence of a biological norm: it is another norm but one which is, comparatively speaking, pushed aside by life.” (Canguilhem 1991, p. 197) In the *Essay* (Canguilhem 1991, p. 206), see also on this point the difference between two modes of life, characterized by normal constants having propulsive value (“truly normal by virtue of their normativity”) and having a repulsive value (“expressing the death of normativity” in those living beings).

11 I will draw on the contribution of the two authors to present this question.

12 The passage is in Canguilhem (1991, p. 263), but I am citing the translation proposed by Gayon (1998, p. 315).

13 Note that in this text from 1977 Canguilhem uses the term “normality” with a meaning that seems to correspond to “normativity” of his earlier writings.

14 My translation. “...S’agissant d’existants dont la vie est une différence de valeur d'avec la mort (...)” (Canguilhem, 2009, p. 168).

15 “…La qualité de la relation au milieu qui permet à ces vivants de permettre, à leur tour, par les variations individuelles de leurs descendants, de nouvelles formes de relations à un nouveau milieu, et ainsi à la suite.” (Canguilhem 2009, p. 167).

16 On Canguilhem’s approach about questions of inheritance and Lamarckism, see for example Méthot (2020).

17 “An attribute within the order of values” translates “un caractère dans l’ordre des valeurs”. Interestingly, Gayon (1998) rather translates: “a character in the kingdom of values” (p. 317).

18 “The relation between the living and the milieu establishes itself as a debate (*Auseinandersetzung*), to which the living brings its own proper norms of appreciating situations, both dominating the milieu and accommodating itself to it. This relation does not essentially consist (as one might think) in a struggle, in an opposition. That applies to the pathological state. A life that affirms itself against the milieu is a life already threatened. (…) A healthy life, a life confident in its existence, in its values, is a life of flexion, suppleness, almost softness.” (Canguilhem 2008, p. 113).

19 Note that, on the notion of adaptation, Canguilhem makes a distinction between: (a) Adaptation as a specialization for a specific task and (b) Adaptation as independence from the environment (Canguilhem 1991, p. 262) The first kind of adaptation, contrary to the second, is threatened by the inconstancies of the environment.

20 It is worthwhile to mention that the active role of the organism relative to environment was already acknowledged by Darwin, namely in his work on earthworms (Darwin 1881).

21 I agree with Sholl (2020) that the point is not to read Canguilhem as a “precursor”. Rather the idea is to consider Canguilhem as a fruitful author to think with, because his work deals with some issues and concepts that are at the centre of contemporary debate.

22 Daniel McShea agrees on this point (McShea, personal communication).

23 Maybe a parallel could be done here about the “levels” at which the goodness of the relationship is considered. In “ecological fitness” and in Canguilhem’s normativity discussed for the categories of normal and pathological, the goodness concerns the relationship between the organism and the environment. But in “evolutionary fitness” and in Canguilhem’s normativity discussed in an evolutionary perspective, there seems to be a displacement towards considering what is good for a lineage. On this point cf. Sholl (2020).

24 Brandon (1990) has pointed out that there is a trade-off between the generality of the definition of fitness (the possibility to apply it to every organism, in all environments) and its applicability (the possibility to actually measure fitness). I thank Daniel McShea for drawing my attention on this point, which would merit further analysis.

25 My translation. “L’existence n’a pas de la valeur, elle n’est pas la valeur, mais l’existence prend valeur en permettant à la valeur de valoriser.” (Canguilhem, Unpublished work, fol. 132).

26 My translation. “Le besoin crée la valeur”, in Limoges (2015, p. 36).

27 Canguilhem, Unpublished work, “La valeur et la polarité du jugement”.

28 My translation. “En fait, les valeurs sont au niveau de la polarité: elles sont cette polarité même.” (Canguilhem, Unpublished work, “La valeur et la polarité du jugement”).

29 However, it should be noted that this dichotomy has been questioned in analytic metaethics by Korsgaard (1983), who claims that two orthogonal distinctions should be made: intrinsic/extrinsic values (concerning the source of goodness, good in itself or in virtue of some relational properties) and instrumental/final values (concerning the evaluation as a means or as an end).

30 Canguilhem, Unpublished work, “La valeur et la polarité du jugement”.

31 My translation. “I (...) il n’est pas exact que toute valeur soit d’utilité, car l’utilité c’est strictement la valeur d’un moyen.” (Canguilhem 2015, p. 83).

32 “Or il est constant—sinon normal—que les hommes s’intéressent d’abord aux moyens, ou du moins à ce qu’ils jugent tels *sans voir qu’il n’y a de moyens que par des fins* (…)” (Canguilhem 2015, p. 83).
